# Periodontal Pathogens as Risk Factors of Cardiovascular Diseases, Diabetes, Rheumatoid Arthritis, Cancer, and Chronic Obstructive Pulmonary Disease—Is There Cause for Consideration?

**DOI:** 10.3390/microorganisms7100424

**Published:** 2019-10-09

**Authors:** Denis Bourgeois, Camille Inquimbert, Livia Ottolenghi, Florence Carrouel

**Affiliations:** 1 Laboratory “Systemic Health Care”, EA4129, University Claude Bernard Lyon 1, University of Lyon, 69008 Lyon, France; denis.bourgeois69@orange.fr (D.B.); camille.inquimbert@gmail.com (C.I.); 2 Department of Public Health, Faculty of Dental Medicine, University of Montpellier, 34080 Montpellier, France; 3 Department of Oral and Maxillo-facial Sciences, Sapienza University of Rome, 00185 Rome, Italy; livia.ottolenghi@uniroma1.it

**Keywords:** noncommunicable disease, periodontal disease, periodontal bacteria, cancer, cardiovascular diseases, diabetes, pulmonary diseases, rheumatoid arthritis

## Abstract

Cardiovascular diseases, chronic obstructive pulmonary diseases, diabetes, rheumatoid arthritis, and cancer are the most common noncommunicable diseases (NCDs). These NCDs share risk factors with periodontal disease (PD), a preventable risk factor linked to lifestyle. The discussion regarding the association between these chronic diseases is more complex. There is still a significant knowledge gap particularly of the causal relationship between PD and NCDs. In this paper, we present fundamental knowledge of the mechanisms and roles of putative periodontal bacteria to gather several hypotheses, evidence that clinical studies thus far have not produced. Although the causal hypotheses are not yet clearly established on a biological basis, prevention and prophylactic measures are recommended to prevent even the possibility of such potential risk factors.

## 1. Introduction

The Sixtieth World Health Assembly (WHA60.17) highlighted in 2007 the link between oral health, general health and the quality of life. It explained that the prevention and treatment of chronic diseases requires the establishment of prevention programs for oral disease and oral health promotion [1]. This assembly, with the support of the World Dental Federation, advocated for member states to introduce rules promoting oral health directly into their policies for the treatment and prevention of chronic noncommunicable and communicable diseases [2,3,4].

Cardiovascular diseases, chronic obstructive pulmonary diseases, rheumatoid arthritis, diabetes, and cancer are the most common noncommunicable diseases (NCDs). These NCDs and periodontal disease (PD) have identical but preventable risk factors linked to lifestyle [5]. Thus, the link between several oral diseases and NCDs is due to the fact that these diseases share risk factors [6]. Many general pathologies are characterized by oral manifestations, which increases the risk of oral pathology. Conversely, oral diseases are a risk factor for general pathologies. This approach, based on common determinants, has received significant support from research and evidence-based approaches. If the concept of common risk factors is indisputable, the interrelationship between oral diseases and NCDs is proven by evidence [7].

The oral cavity of humans contains more than 700 bacterial species that are able to penetrate the digestive and respiratory tracts [8]. The oral microbiota is a key factor in the protection against the colonization of extrinsic pathogens that could impact systemic health [9]. However, the imbalance of the ecosystem, which can be caused by a weak immune system, leads to a challenge for oral and systemic health. The ecological conditions of these habitats are constantly changing, so ecosystems are subject to frequent variations.

The oral microbiome is a key factor in health or disease [10]. Its modification contributes to oral and systematic diseases [11]. Moreover, dysbiosis of the oral ecosystem is associated with systemic diseases, such as cardiovascular diseases, cancers, and diabetes [12].

A systematic literature search was conducted, fulfilling PRISMA criteria (Preferred Reporting Items for Systematic reviews and Meta-analyses). Electronic research was organized in Pubmed, Embase, and Cochrane databases. No language filters were implemented. The research was performed from 2000 to April 2019. The following MeSH and non-MeSH search terms were used in order to encompass every type of periodontal pathogens and cardiovascular diseases, diabetes, rheumatoid arthritis, cancer, and chronic obstructive pulmonary disease: (“periodontitis”[MeSH Terms] OR “periodontitis”[All Fields]) AND (“Pathogens”[Journal] OR “pathogens”[All Fields]) AND (“diabetes mellitus”[MeSH Terms] OR (“diabetes”[All Fields] AND “mellitus”[All Fields]) OR “diabetes mellitus”[All Fields] OR “diabetes”[All Fields] OR “diabetes insipidus”[MeSH Terms] OR (“diabetes”[All Fields] AND “insipidus”[All Fields]) OR “diabetes insipidus”[All Fields]) AND (“cardiovascular diseases”[MeSH Terms] OR (“cardiovascular”[All Fields] AND “diseases”[All Fields]) OR “cardiovascular diseases”[All Fields]) AND (“arthritis, rheumatoid”[MeSH Terms] OR (“arthritis”[All Fields] AND “rheumatoid”[All Fields]) OR “rheumatoid arthritis”[All Fields] OR (“rheumatoid”[All Fields] AND “arthritis”[All Fields])) AND (“neoplasms”[MeSH Terms] OR “neoplasms”[All Fields] OR “cancer”[All Fields]) AND (“pulmonary disease, chronic obstructive”[MeSH Terms] OR (“pulmonary”[All Fields] AND “disease”[All Fields] AND “chronic”[All Fields] AND “obstructive”[All Fields]) OR “chronic obstructive pulmonary disease”[All Fields] OR (“chronic”[All Fields] AND “obstructive”[All Fields] AND “pulmonary”[All Fields] AND “disease”[All Fields]). The selection procedure was performed by two reviewers, who evaluated the titles and abstracts of the articles identified in the electronic databases. Finally, 98 studies were included in this review.

In this review, fundamental knowledge of the mechanisms and roles of putative periodontal bacteria on the potential development of the five main noncommunicable diseases—cancer, cardiovascular diseases, diabetes, pulmonary diseases, rheumatoid arthritis—are presented.

## 2. Periodontal Disease

Periodontal disease (PD), classified by the World Health Organization (WHO) as a noncommunicable disease (ICD-10, K.053), is a microbe-induced inflammatory and multifactorial chronic immunologic disease [13]. Periodontitis is a polymicrobial infection due to an increase in pathobionts within the microbiota [14]. The initially synergistic microbiota gradually becomes dysbiotic [15]. Bacterial accumulation around the tooth on gingival or subgingival level gives rise to an inflammatory response in the tissues surrounding the teeth. The inflammation of the gum leads to the destruction of the alveolar bone and loss of gingival attachment to the teeth. PD is a progressive disease that ranges from gingivitis (reversible inflammation) to periodontitis (irreversible inflammation). The evolution of PD is correlated with several modifiable and unmodifiable risk factors [16]. PD has been the subject of many studies for many years within the scientific community that aimed to identify the links that may exist among systemic diseases [17]. Several bacterial species associated with periodontitis have been suggested to be involved in the pathogenesis of some systemic diseases [18]. However, because of the complexity of these periodontal-systemic associations and conflicting scientific reports, most of the associations remain speculative [19].

Periodontopathogenic bacteria according to their pathogenicity and properties are organized into complexes that are closely interrelated [20]. The bacteria from the green, purple, and yellow complexes are named “early colonizers” because they are able to adhere to the pellicle. The orange complex comprises putative periodontal pathogens, such as *Fusobacterium nucleatum (F. nucleatum)*, that generally appear after the early colonizers are established [21]. These bacteria, classified as moderately pathogenic, associate with other periodontal bacteria to form the basis for the colonization of the sulcus. The highly pathogenic bacteria of the red complex, which include *Porphyromonas gingivalis (P. gingivalis*), *Treponema denticola (T. denticola)*, and *Tannerella forsythia (T. forsythia)*, are the most important pathogens in adult PD [22]. The recent “Keystone-Pathogen Hypothesis” (KPH) considers that certain bacteria in low quantities, such as *P. gingivalis*, can act on the host immune system and convert the microbiota from symbiotic to dysbiotic to provoke inflammatory disease [16]. The host immune system could be modified. In fact, *P. gingivalis* could act on the immune system of the host in three different ways. This pathogen may alter the Toll-like receptor (TLR) response, subvert interleukin-8 (IL-8), or alter the complement system [23,24]. First, during inflammatory process, *P. gingivalis* lipopolysaccharide (LPS) expression increases which reduces the TLR4 response and could facilitate survival and multiplication of the entire microbial community [25]. Then, *P. gingivalis* can block production of IL-8, which is produced by gingival epithelial cells in response to other bacteria, by secreting a serine phosphatase that inhibits the synthesis of IL-8 [26]. This process delays the recruitment of neutrophils and could facilitate initial microbial colonization of the periodontium [27]. Other bacteria from the red complex such as *T. denticola*, are also able to manipulate the IL-8 response of the host [28]. Finally, *P. gingivalis* is able to avoid complement-mediated detection by producing gingipains (membrane bound and soluble arginine-specific cysteine proteinases). Gingipains cleave complement factors C3 and C5 into active fragments C5a (cell activator) and C3b (phagocytosis enhancer) and degrade them [29]. The increasing of C5a leads to an increased activation of the C5a receptor on leukocytes [29]. C5a receptor is involved in crosstalk with TLR2, which is activated in parallel by *P. gingivalis* surface ligands. While this crosstalk leads to increased inflammation, it impairs the killing capacity for leukocytes [30].

The pathogenicity of these bacteria is significantly increased following the production of various enzymes and toxins. The attachment loss and an increase in pocket depth are due to bacteria belonging to the orange complex. Through their metabolism, these bacteria also create living conditions for red complex bacteria that are strict anaerobes and thus allow them to colonize the sulcus. The presence of bacteria from the red complex and the *Aggregatibacter actinomycetemcomitans* (*A. actinomycetemcomitans*) complex is the witness of the final colonization phase. The pathogenesis of PD results from the complex interaction between periodontal pathogens and the host immune response, controlled by environmental and genetic factors. Another concept named the “ecological plaque hypothesis” considers that groups of bacteria, such as the bacteria from the red complex, can create an ecosystem capable of inducing periodontal disease [31]. The pathogenesis of PD could be the result of a dysbiosis in the microbiota caused by ecological stress due to the enrichment of several pathogens [30].

Therefore, the inflammation and tissue destruction in the case of periodontitis is not only due to the presence of some periodontopathogens such as bacteria from the red complex but to the dysbiosis of the oral microbiota that they induce [32].

## 3. The Invasion Process by Periodontal Bacteria

**Figure 1:**
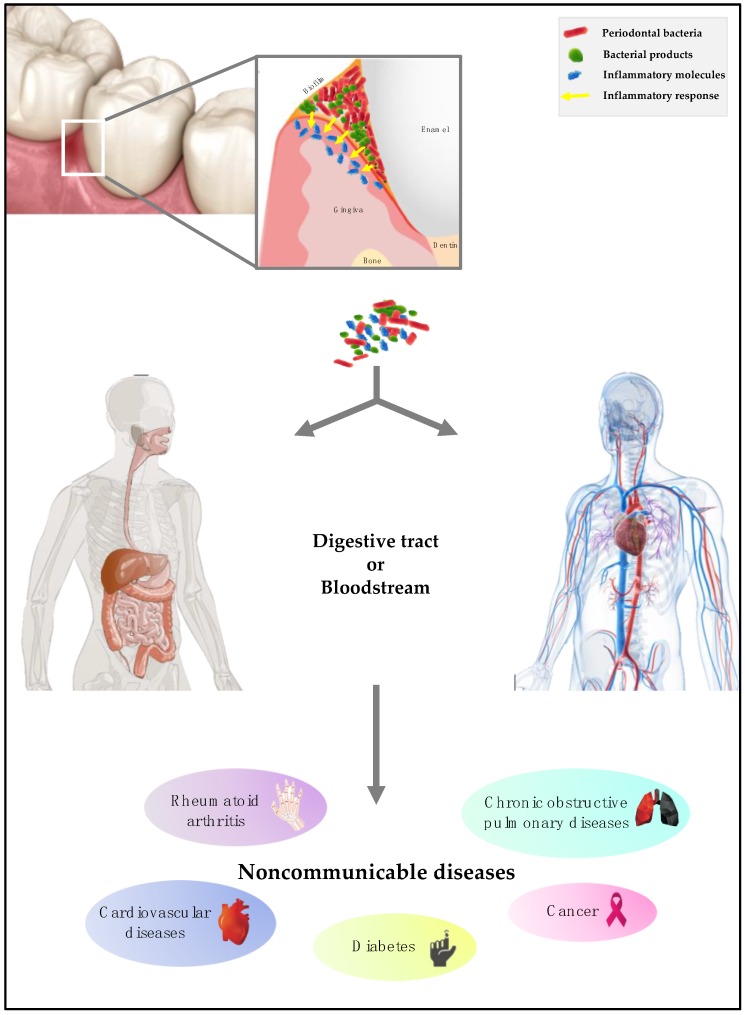
Process of invasion of the body by periodontal bacteria.

Oral bacteria, bacterial products, and inflammatory molecules can invade the human body in two main ways: (i) the bloodstream or (ii) the digestive tract (Figure 1).

First, bloodstream invasion is possible because anatomically, the periodontal pockets are close to the bloodstream. Their contents (periodontal bacteria, bacterial products, immunocomplexes, and mediators of inflammation) will therefore be able to diffuse and reach different sites of the human body [33]. Periodontal pathogenic bacteria that have the property of being mobile will be able to migrate and invade the epithelium and then the connective tissue [34] before reaching the bloodstream. In patients with periodontitis, gingival ulceration is the major cause of bacteremia. Bacterial products such as exotoxins and endotoxins are also able to reach the bloodstream and thus diffuse to exert their toxicity at a distance. Thus, endotoxins are lipopolysaccharides of the outer membrane of Gram-negative bacterial cells responsible for numerous pathologies [35]. Pro-inflammatory molecules such as interleukin (IL) 1β, IL-6, or tumor necrosis factor (TNF) will reach the systemic circulation, triggering a response in other tissues such as the liver [36].

Finally, by alimentary dissemination, oral bacteria, bacterial products, and inflammatory molecules, reach the digestive tract including the stomach. During digestion, the oral bacteria will migrate into the stomach. Only those that resist the acidic pH of the stomach will survive and multiply in the gastrointestinal tract [37,38]. Thus, the oral microbiota and the microbiota of the large intestine have similarities in 45% of subjects with PD [39]. Previous studies demonstrated that the modification of the oral microbiota due to oral disease is linked to the dysbiosis of the gut microbiota [40]. This correlation between the oral microbiota and the intestinal microbiota is due to daily activities (e.g., swallowing) that promote the transport of bacteria from the oral cavity to the gastrointestinal tract. However, the precise mechanism of invasion must be determined [41].

## 4. Periodontal Pathogens and Diabetes

Diabetes has a dual association with PD [42]. Identified as chronic pandemic diseases, PD and type 2 diabetes (T2DM) are also risk factors for cardiovascular complications [43]. The hypothesis of a causal relationship between the imbalance of the periodontal microbiota and the incidence of metabolism disease is advanced [44]. Periodontal infection and subsequent inflammation increase insulin resistance and negatively affect glycemic control. This is partly explained by the increase in the level of systemic pro-inflammatory mediators—cytokines in particular, which exacerbate insulin resistance—and by the chronic bacteremia that accompanies periodontitis. In the case of PD, several inflammatory molecules, such as IL-1β, IL-6, IL-8, LPS, TNF-α, and prostaglandin (PG) E2, are liberated. These molecules are able to interact with free fatty acids, lipids, and advanced glycation end products, all of which are characteristic of diabetes. Thus, some intracellular pathways associated with insulin resistance are impacted, such as the I-kappa-B (IκB), I-kappa-B kinase-β (IKKβ), nuclear factor-kappa B (NF-κB), and the protein c-Jun N-terminal kinase (JNK) axes. JNK promotes insulin resistance by phosphorylating serine residues in the insulin receptor substrate-1. The counter-regulatory phosphorylation of serine and threonine inhibits the insulin receptor signaling that normally occurs through a tyrosine kinase cascade. Concerning IKKβ, the insulin resistance results from the activation of NF-κB transcription. Activation of IKKβ leads to the phosphorylation of IκB, a cytosolic inhibitor of NF-κB. Phosphorylation by IKKβ targets IκBα for proteasomal degradation, which liberates NF-κB for translocation into the nucleus, where it initiates the transcription of various genes involved in insulin resistance, such as growth factors, cytokine genes (IL-1, IL-6, IL-8, and TNF-α), adhesion molecules, and proteins in the acute phase. The activation of these inflammatory pathways in hepatocytes, endothelium cells, immune cells (monocytes or macrophages), muscle cells, and adipocytes promotes and contributes to an increase in overall insulin resistance, which makes it difficult to perform metabolic regulation in individuals with both PD and T2DM [45].

**Figure 2:**
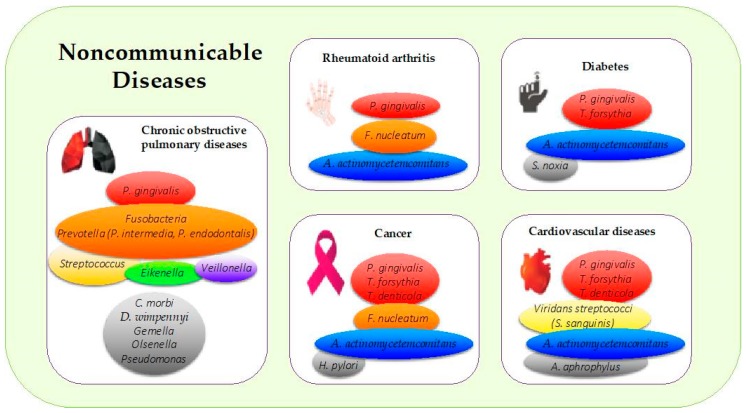
Periodontal pathogens implicated in the most common noncommunicable diseases. The colors in boxes refer to (i) the colors of the Socransky complexes for the purple, green, yellow, orange, and red colors, and (ii) other periodontal bacteria for the gray color. *A. actinomycetemcomitans: Aggregatibacter actinomycetemcomitans*; *C. morbi*: *Cantonella morbi; D. wimpennyi: Dysgonomonas wimpennyi; F. nucleatum: Fusobacterium nucleatum; H. pylori: Helicobacter pylori; P. gingivalis*: *Porphyromonas gingivalis*; *S. noxia*: *Selenomonas noxia; S. sanguinis*: *Streptococcus sanguinis*; *T. denticola*: *Treponema denticola*; *T. forsythia*: *Tannerella forsythia.*

The consequences of periodontal microbiota disbalance were also significant at the systemic level, with metabolic modifications connected to diabetes [46]. Additionally, a discordance in the microbial profile between chronic periodontitis patients with and without T2DM has been emphasized. The strength of evidence is robust for *T. forthysia*, which is related to being less frequent in the T2DM-PD group, followed by the lowest evidence for other pathogens such as *Aggregatibacter actinomycetemcomitans* and *P. gingivalis* [47] (Figure 2).

The role of oral bacteria in adiposity was also demonstrated. *Selenomonas noxia* (*S. noxia*) represented more than 1.05% of the total bacterial in 98.4% of overweight women [48]. *S. noxia* could be a predictive marker of obesity given its sensitivity and specificity. This leads to the relevant hypothesis of whether the bacteria of the oral sphere are implicated in the process that is likely to lead to obesity. The role of periodontopathogenic bacteria in this pathology is yet to be established. However, animal studies have demonstrated that the modification of the intestinal microbiota by taking specific nutrients, prebiotics, or natural antibiotics could modify satiety and insulin resistance and thus allow better control of diabetes [49,50].

## 5. Periodontal Pathogens and Cardiovascular Diseases

The hypotheses from the literature strongly argue for an increased impact of long-term periodontitis on the main noncommunicable diseases.

Periodontal infections are strongly associated with the development of atherosclerosis [51]. The systemic inflammatory or immune response to periodontal infection may increase cardiovascular risk. Additionally, pathogens from the mouth can cross the gingival epithelio-conjunctive barrier as well as the vascular endothelium and enter atherosclerotic plaques via the bloodstream, which could promote an inflammatory or immune response within the atherosclerotic plaque [52].

According to Aarabi and colleagues, four different mechanisms could explain the link between oral disease and the pathological process of atherosclerosis. First, an oral bacterium reaches the bloodstream following bacteremia. Second, during an oral disease, mediators of inflammation are released and can enter the bloodstream. Third, following exposure to components of oral pathogenic bacteria, an autoimmune reaction against host proteins occurs. Finally, some oral pathogens produce toxins with pro-atherogenic action [53].

*P. gingivalis* can intensify atherosclerosis after oral-hematogenous spread due to bacteremia. In its presence, endothelial cells activate certain adhesion molecules, thus increasing the likelihood of macrophage diapedesis and the subsequent conversion to foam cells and further atheroma progression. PGE2, TNF-α, and IL-1b produced locally at the periodontal pockets in response to PD bacteria will end up in the bloodstream, causing a disproportionate increase in the local tissue innate immune response. However, the mechanism of the active invasion of endothelial cells by *P. gingivalis*, likely to adjust the inflammatory response of these cells, remains unclear [54,55].

The association of coronary heart disease and PD may be due to a trait underlying response, which puts a person at high risk of codeveloping periodontal diseases and coronary artery disease. Once established, PD provides a bioburden of endotoxin (lipopolysaccharide) and inflammatory cytokines, especially thromboxane A2, PGE2, IL-1α, and TNF-β, which serve to initiate and exacerbate atherogenesis and thromboembolic events [56]. In atherosclerotic plaques, various periodontal bacteria have been identified [57]. Moreover, in mouse models, *P. gingivalis* increases the progression of inflammatory plaque accumulation in the innominate arteries with the accumulation of inflammatory mediators and cholesterol esters [58]. Serum IgA antibodies to *P. gingivalis* are significantly higher in Chinese patients with myocardial infarct [59].

PD is correlated with an increased risk of future myocardial infarction. However, a recent meta-analysis from observational studies could not establish a causative relationship between PD and myocardial infarction. Additional investigations are recommended [60]. Analysis of thrombi collected by aspiration during interventions on the coronary arteries of patients who had a myocardial infarction showed 19.7% *A. actinomycetemcomitans*, 3.4% *P. gingivalis*, and 2.3% *T. denticola* [61]. Antibody levels against four major periodontal pathogens, *P. gingivalis*, *A. actinomycetemcomitans, T. forsythia*, and *T. denticola*, are related to an increased relative risk of myocardial infarction [62] (Figure 2). Other studies highlight the important role of oral *Viridans streptococci* in the development of myocardial infarction [63,64,65].

Infection with *P. gingivalis* after myocardial infarction in mice enhanced myocardial high mobility group box 1 (HMGB1) expression. HMGB1 is a nuclear protein released from necrotic cells and capable of inducing the inflammatory response. There is a possible relationship between PD and postinfarction myocardial inflammation through HMGB-1 [66].

Infection with *P. gingivalis* during myocardial infarction generates a prejudicial part in the recuperation procedure of the infarcted myocardium by penetration and invasion of *P. gingivalis* into the myocardium, thus favoring programmed cell death and the matrix metalloproteinase (MMP) 9 action of the myocardium, which successively produces cardiac rupture [67].

Clinical studies particularly suggest a direct relationship between the severity of periodontal conditions and left ventricular hypertrophy. In animal transverse aortic constriction models, *A. actinomycetemcomitans*, a Gram-negative bacterium that is considered an etiologic agent in endocarditis, clearly improved cardiac hypertrophy with matrix MMP-2 activation [68].

Control of chronic inflammation caused by periodontitis may positively impact the treatment of myocardial hypertrophy, decreasing the risk of acute myocardial infarction [69].

The risk of stroke, described by a meta-analysis of cohort studies, was significantly increased by the presence of periodontitis [70]. PD was significantly correlated with cardioembolic and thrombotic stroke subtypes. Regular dental care utilization was associated with a lower adjusted stroke risk [71]. Pussinen and colleagues have established that *P. gingivalis*, a Gram-negative anaerobic bacterium, may especially be correlated with stroke [72].

Experimental rats with periodontitis and stroke may have impaired endothelial function in gingival tissues. These studies show that the disruption of vascular function in oral microcirculation may be generated by the fundamental interaction between the oxidative stress induced by PD and nitric oxide, similar to the interactions existing in stroke cases [73]. Reducing the risk of stroke therefore requires the management of PD. It also involves daily individual prophylaxis of oral health, whose purpose is the disruption and removal of the biofilm. It is necessary to control the risk of chronic inflammation, which can lead to tragic consequences such as stroke [74]. These are arguments, as recommended by the WHO, for developing intersectoral approaches to chronic disease control in an alliance of general practitioners, dentists, nurses, and specialists [75].

*A. actinomycetemcomitans* and *Aggregatibacter aphrophilus* belong to the HACEK (*Haemophilus*, *Aggregatibacter*, *Cardiobacterium*, *Eikenella*, *Kingella*) group of Gram-negative bacteria, a recognized cause of infective endocarditis (Figure 2). HACEK organisms are a part of the normal microbiota of the oral and upper respiratory tract in humans. However, these bacteria are implicated in 1% to 3% of all infective endocarditis [76]. *A. actinomycetemcomitans* is also implicated in the etiology of aggressive periodontitis and generates many virulence factors, such as leukotoxin (repeats-in-toxin protein), which kills human immune cells [77,78].

*Streptococcus sanguinis* (*S. sanguinis*), a commensal bacterium, profuse in periodontitis, is recognized as an origin of infective endocarditis [79]. Its fimbriae and adhesin facilitate its initial attachment on the tooth. Then, the production of glucans and eDNA promotes the maturation of *S. sanguinis* biofilm. After accessing the heart, *S. sanguinis* must then adhere to the endocardium. Considering the impact of biofilm formation on adhesion in the oral cavity, it would be conceivable that biofilm formation might be significant for adhesion to endocardial surfaces as well. Indeed, endocarditis is frequently regarded as a model of a biofilm-mediated disease [80,81]. However, studies have demonstrated that *S. sanguinis* endocarditis causation is not dependent upon biofilm formation [79]. Therefore, in contrast to this situation in the oral cavity, there is as yet no evidence that biofilm formation is important for *S. sanguinis* in the cardiac environment in relation to infective endocarditis [79].

## 6. Periodontal Pathogens and Chronic Obstructive Pulmonary Disease

While respiratory diseases are among the leading causes of death in the world, tobacco is the major risk factor [82]. Nearly half of all chronic obstructive pulmonary disease (COPD) deaths are attributable to smoking; 80–90 % of cases of COPD can be avoided by refusing tobacco [83,84]. Periodontal pathogens and inflammatory cytokines can generate systemic inflammation, which can take part in the pathogenesis of chronic obstructive pulmonary disease (COPD) [85]. There are similarities in the disease mechanisms—sustained neutrophil inflammation, dysfunctional neutrophil behaviors, and connective tissue loss—that imply a common pathophysiology and confirms the association in clinical evidence that resulted from the meta-analyses [86].

The processes that are suggested to associate periodontal diseases and COPD include the overspill of topically generated inflammatory mediators into the systemic circulation or bacteremia of oral or pulmonary origin that accelerates acute response, mechanical aspiration of oral content in the tree respiratory, and reactive oxygen species and cytokines released by systemic neutrophils at distant sites [85]. Oral symbiosis requires professional management and regular visits to reduce the virulent bacterial load, and consequently, a reduction in the incidence or severity of the COPD occurs. Daily individual oral hygiene is a major factor that decreases the risk of disease among subjects with respiratory illness [87].

The PD and COPD association do not necessarily require bacterial aspiration through the respiratory tract. Local cytokines and other active molecules produced by periodontal inflammation penetrate into the systemic circulation. The endothelium and circulating immune cells are thus activated. Both contribute to the inflammatory burden by the liberation of activated and destructive mediators. This process also takes place in the lungs, initiating pulmonary inflammation. Periodontal bacteria move in gingival vasculature via microulcerations in the epithelium, permitting hematogenous dispersion of bacteria and inflammatory mediators [88].

Among the oral bacterial species involved in bronchopulmonary pathologies, *Streptococcus*, *Veillonella, Gemella, Porphyromonas, Olsenella*, and *Eikenella* were found [89] (Figure 2). Identified in several studies, *Fusobacteria, Pseudomonas, Prevotella*, and *Streptococcus* make up the pulmonary microbiome of COPD [90] (Figure 2). Both chronic periodontitis and COPD are associated with an increase in *P. intermedia, Catonella morbi, Dysgonomonas wimpennyi*, and *Porphyromonas endodontalis* as well in the genera *Dysgonomonas*, *Desulfobulbus*, and *Catonellas*. Thus, active care of periodontitis, with the goal of reducing virulent bacteria, should have a direct impact and benefit on COPD subjects. However, more longitudinal studies are required to validate this hypothesis [91].

## 7. Periodontal Pathogens and Rheumatoid Arthritis

Strong epidemiological, serological, and clinical associations have been observed between rheumatoid arthritis (RA) and periodontitis [92]. The presence of PD might contribute to the progression of RA, while RA might have little effect on accelerating the development of PD. RA and periodontitis share many common pathological features, such as chronic inflammation induced by pro-inflammatory cytokines, connective tissue breakdown, and bone erosion [93]. Mutual in both diseases, there is a growing diversity of MMPs and cytokines. However, despite this evidence showing a link between rheumatoid arthritis and periodontitis, the exact mechanisms involving this association have not been fully elucidated [94].

The periodontal pathogen *A. actinomycetemcomitans* has been recognized as a bacterial trigger for RA, providing a link between autoimmunity and periodontal diseases. Indeed, the oral pathogens may trigger the production of disease-specific autoantibodies and arthritis in susceptible individuals. Periodontitis is characterized by the presence of citrullinated autoantigens that are primary immune targets in RA. *A. actinomycetemcomitans* provokes activation of the citrullinating-enzyme process in neutrophils and thus autoantigen output [95,96].

Most of the studies have shown the presence of oral bacteria in patients with RA, highlighting *P. gingivalis* and *F. nucleatum* [97] (Figure 2). Some periodontal pathogens, such as *A. actinomycetemcomitans* and *P. gingivalis*, may take part in RA autoantibody production through direct posttranslational alteration of proteins or indirectly through impacting neutrophil-mediated neo-epitope generation. *P. gingivalis* is often found in the synovial joints of RA. *F. nucleatum* has been detected in synovial fluid and in plaque samples of patients with joint pathologies [98]. Periodontal bacteria that overrun the bloodstream may also take part in chronic inflammatory reactions and the production of antibodies [99].

Hypotheses have suggested that chronic periodontitis generates local and constant high levels of microparticles that spread into the bloodstream and are considered inflammatory biomarkers or mediators responsible for distant cell signaling and regulation [100]. It could establish a demonstration of the increased risk for chronic disease in adults with PD [51]. It is now validated that the association between infectious pathogens and autoimmune arthritis is more complicated than the one pathogen-one disease process that consolidates the conceptual frame for Koch’s postulates. Immuno-pathological similarities between these two conditions induce a greater risk for patients with periodontitis to develop RA [101,102].

A better quality of life for patients with RA requires consideration of the inflammatory process of periodontal disease with care to prevent, reduce, or cure periodontitis [103,104]. Reduced systemic inflammation might contribute to a better clinical outcome of RA [93].

## 8. Periodontal Pathogens and Cancer

The human microbiota may play a role in carcinogenesis. At this time, biological hypotheses and animal and human studies data corroborate the significant associations between PD and pancreatic, neck, and head cancers and the risk of lung cancer [105]. Large cohort studies validated by meta-analyses confirm the evidence of some positive associations between PD and total cancer risk, particularly for head and neck, pancreas, and lung cancers. Unfortunately, the dispersion of the clinical parameters used for the definition of periodontitis and the difficulties of adjustment for smoking status, lacking in some studies, penalize the impact of the results of eligible studies for meta-analyses [105].

The biological hypothesis for the relationship between PD and carcinogenesis includes chronic inflammation, attendance of periodontal pathogens, and a reservoir for potential carcinogenic factors (e.g., Human papillomavirus). As specified by Mantovani and colleagues, nonsteroidal anti-inflammatory medication decreases the incidence of certain cancer categories while reducing mortality [106]. Furthermore, in experimental animal models, inflammatory cells, cytokines, and chemokines are present in the microenvironment of all tumors, and in humans, inflammatory cells, cytokines, and chemokines are present from the early stages of tumor development; the overexpression of inflammatory cytokines may promote tumor development.

Smoking and alcohol consumption, which are clearly identified risk factors, can be considered classic confounders. However, after adjustment, the association of causality between periodontitis and the risk of oral cancers and/or oropharynx remains significant [93,107]. *P. gingivalis* infects the epithelium of the esophagus of esophageal squamous cell carcinoma (ESCC) subjects, which establishes a relationship between infection with *P. gingivalis* and the progression of ESCC and proposes that *P. gingivalis* infection could be a biomarker for this disease [108]. In this context, the eradication of virulent periodontal bacteria, common to PD and ESCC, could probably contribute to reducing the global burden of these cancers [109].

The association between periodontitis and pancreatic cancer (PC) is well documented in the literature. These are targeted studies of periodontal bacteria that may play a key role in the pathogenesis of PC [110]. A current cohort survey, using direct bacterial DNA quantification from the saliva of subjects recovering years prior to diagnosis, identified associations between *A. actinomycetemcomitans*, *P. gingivalis*, and pancreatic cancer [111,112] (Figure 2). *F. nucleatum*, a member of the orange complex, has been associated in many studies with colorectal cancer [113]. However, it is uncertain whether this pathogen is identified at higher levels in the bowel as an outcome of periodontitis.

The dysbiosis of the periodontal microbiota is associated with several chronic diseases, such as gastrointestinal system diseases. Inflammatory bowel disease was one of the first to be highlighted. Scientific advances have increased the level of correlation evidence between PD and gastrointestinal cancers and liver cirrhosis [11]. The risk of developing precancerous gastric lesions is increased in the presence of potentially virulent periodontal factors and with the complex and diverse nature of the oral microbiota [114]. In periodontitis, significant levels of the colonization of pathogens, members of the red complex of Socransky, and *A. actinomycetemcomitans*, are correlated with an increased risk of gastric precancerous lesions. An increase in the DNA of selected bacteria (*P. gingivalis, T. forsythia, T. denticola*, and *A. actinomycetemcomitans*) is associated with increased levels of PD indices [115].

The most significant risk component of noncardiac gastric cancer, found in all other areas of the stomach other than the upper stomach, is *Helicobacter pylori* (*H. pylori*), potentially present in the oral microbiome (e.g., saliva and dental plaque). The pathogen may be dissolved orally. The infection rate is essentially a function of hygiene condition as well as the degree of antibiotic use [116,117,118,119]. Initial results from clinical trials have shown that *H. pylori*-positive dyspeptic patients may benefit from periodontal therapy.

Knowledge of the processes of *H. pylori*-induced carcinogenesis remains limited, although inflammation is a frequently cited hypothesis. Inflammation could be responsible for cancer by increasing the production of free radicals, increasing apoptotic and necrotic epithelial cell death and augmenting cell proliferation [120]. With this hypothesis, the validation of a relationship between *H. pylori* and gastric cancer has led to a better understanding of the gastrointestinal microbiome and novel strategies for the prevention of individual gastric cancer [121]. *H. pylori* is closely associated with periodontitis with the potential to adhere to certain orange and red complex bacteria, such as *Fusobacterium* species (*F. nucleatum*), *P. gingivalis*, and *T. forsythia* [122,123]. As the amount of these pathogens is increased in periodontitis, it is more probable that mature biofilm may shelter *H. pylori* by interacting with these bacterial species [58].

The evidence of the existence of the same *H. pylori* strain in plaque and stomach samples may clarify the role of dental plaque as a sanctuary site for *H. pylori* and as a source of reinfection after eradication, which is more difficult to eradicate from plaque than from the stomach [124].

## 9. Conclusions

This paper alone cannot answer the question of the causal link between periodontal bacteria and the main noncommunicable diseases that are CVD, diabetes, RA, cancer, and COPD.

There is, however, some hypotheses that periodontal pathogens contribute to the increased risk of NCDs by marginalizing the impact of conventional risk factors such as tobacco use, alcohol, and physical inactivity. The effect of periodontal bacteria on NCDs briefly introduces the putative biological mechanisms involved. Virulent periodontal bacteria almost all have a role in pathogenesis. *P. gingivalis* is the typical example.

Some essential science of the processes and pivotal role of *P. gingivalis* incursion in arterial-cardiovascular disease and cardiovascular cells has been demonstrated. *S. sanguinis* is well known for its role in respiratory diseases and infective endocarditis. In addition, the potential interaction mechanisms described in immunology and molecular biology argue in favor of a potential causality. It is well recognized that the homeostasis of oral microbiome communities is absolutely connected with health factors.

Is the systematic care management of periodontitis indicated to reduce the risk of NCDs? Yes, since by definition any management of PD must be beneficial. Then, there is a convergent web of scientific information in favor of a possible causality. The insufficiency of proof that the treatment of periodontal disease reduces the risk of major adverse NCD events is admissible. For this, large randomized clinical trials are needed. Why has this not been done for 20 years? How long should this be delayed? Between theory and practice, you have to decide. The decision maker must receive specific advice from the scientific community. It is up to us to assess the risk. It should not be neglected and taken into consideration. The insufficient evidence does not compensate for absence of evidence. The methodological difficulties related to the epidemiological study of cohorts, long term, strongly penalize the validation of hypotheses.

Although the association is not fully established and the biological mechanisms are not yet fully understood, there is an obligation of precautionary principles and preventive measures to reduce any potential risk factors. An integrated and collaborative approach to fight common risk factors, as recommended by the WHO, for periodontal disease and chronic diseases is justified. It is important to understand that PD is preventable. A potential gain in oral health from a reduction in a virulent bacterial load can have direct or indirect positive effects on general health and even prevent the possibility of such an association. At least establishing individual prophylaxis in adolescents and young adults, including interdental hygiene, to promote a symbiotic periodontal microbiota throughout their lives, as adequacy in their oral environments remains the highest priority.
